# Assessing the Fungal Simultaneous Removal Efficiency of Carbamazepine, Diclofenac and Ibuprofen in Aquatic Environment

**DOI:** 10.3389/fmicb.2021.755972

**Published:** 2021-12-13

**Authors:** Teddy K. Kasonga, Martie A. A. Coetzee, Ilunga Kamika, Maggy N. B. Momba

**Affiliations:** ^1^Department of Environmental, Water and Earth Sciences, Faculty of Sciences, Tshwane University of Technology, Pretoria, South Africa; ^2^Institute for Nanotechnology and Water Sustainability, School of Science, College of Science, Engineering and Technology, University of South Africa, Roodepoort, South Africa

**Keywords:** ligninolytic enzyme, laccase, manganese peroxidase, lignin peroxidase, biodegradation, carbamazepine, diclofenac, ibuprofen

## Abstract

Unused pharmaceutical compounds (PhCs) discharged into the aquatic environment have been regarded as emerging pollutants due to potential harmful effects on humans and the environment. Microbial bioremediation is considered as a viable option for their removal from wastewater. The aim of this study was to assess the simultaneous removal of carbamazepine (CBZ), diclofenac (DCF) and ibuprofen (IBP) by previously isolated fungi (*Aspergillus niger, Mucor circinelloides*, *Trichoderma longibrachiatum*, *Trametes polyzona*, and *Rhizopus microsporus*). The tolerance to PhCs was conducted by tracking the fungal mycelium mat diameters in solid media and its dry biomass in liquid media, at the drug concentration range of 0.1 to 15 mg/L. The fungal enzymatic activities were determined for lignin peroxidase (LiP), manganese peroxidase (MnP) and laccase (Lac), respectively. The PhC removal efficiency of the fungi was assessed in aerated batch flasks and the drug concentrations and intermediate compounds formation were determined by using SPE-UPLC/MS. A tolerance over 70% was recorded for all the fungi at drug concentration of 0.1 mg/L. Manganese peroxidase was produced by all the fungi with very low amount of LiP, while all the enzymes were produced by *T. polyzona*. The pH of 4.3, temperature 37 ± 1.5°C and incubation time of 6 days were the optimum parameters for the fungal enzymatic activities. The best removal of CBZ (87%) was achieved by *R. microsporus* after 10 days. Between 78 and 100% removal of DCF was observed by all the fungi after 24 h, while 98% of IBP was removed after 2 days by *M. circinelloides*. Only a few intermediate compounds were identified after 3 days and disappeared after 10 days of incubation. This study demonstrated that apart from the basidiomycete*s*, the ascomycetes and zygomycetes are also producers of ligninolytic enzymes and have the ability to biodegrade emerging pollutants such as PhCs.

## Introduction

Thousands of tons of pharmaceutical compounds (PhCs) are annually consumed around the world and residues are discharged with their metabolites into the environment from households, farms, hospitals and pharmaceutical industries (Gadipelly et al., 2014; Ali et al., 2018). These synthetic compounds have complex and aromatic structures, most exhibit abilities to undergo electrophilic substitution reactions, aromaticity and display resonance stability, which decreases their biodegradability and make them more recalcitrant (Gupta et al., 2013; Olicón-Hernández et al., 2017). In addition, their aromatic structures contain complex and multiple substituents including functional groups, for example amine groups on carbamazepine (CBZ), electronegative chlorine and carbonyl groups on diclofenac (DCF) and ibuprofen (IBP), and fluorine groups on ciprofloxacin (da Silva, 2017).

Three PhCs which are frequently detected in the aquatic environment are DCF, IBP (both non-steroidal and anti-inflammatory drugs) and CBZ (anti-epileptic and anticonvulsant drug) (Nakada et al., 2007; Zhang et al., 2008). Researchers have found that the presence of these compounds in water sources induces oxidative stress in fish, while the long-term exposure mainly affects the reproduction parameters (Stancova et al., 2017; Ali et al., 2018). A long exposure can produce adverse risks in the human body as described by Tyumina et al. (2020). A major source of PhCs in the environment is the drug manufacturing industry. Evidence of higher than usual concentrations of PhCs has been reported downstream from a pharmaceutical company. Previous investigators have detected concentrations of up to 1 mg/L of CBZ in the aquatic environment below a pharmaceutical factory (Cardoso et al., 2014). Furthermore, CBZ, DCF, and IBP are amongst the PhCs, which are known to be resistant to biodegradation (Marco-Urrea et al., 2009; Kruglova et al., 2014; Hai et al., 2018).

The discharge of untreated or inadequately treated industrial wastewater containing PhCs into the aquatic ecosystem is one of the most important environmental and health challenges worldwide. The utilization of fungi for treatment of wastewater containing these recalcitrant compounds seems to be an alternative cost-effective solution to conventional techniques. Hatakka and Hammel (2011) have reported that, the extracellular lignin-modifying enzymes which are able to degrade lignin structure are found in basidiomycete fungi, especially white-rot fungi (WRF) including *Trametes versicolor*. Furthermore, Mtui (2012) found these enzymes in ascomycete fungi such as *Aspergillus niger*, and León-Santiesteban et al. (2008) and Pawłowska et al. (2016) in zygomycetes fungi like *Rhizopus oryzae*. Among the ligninolytic enzyme groups, the present study targeted the fungal enzymes which are reported to play a key role in lignin degradation, namely laccases (Lac, E.C.1.10.3.2) and ligninolytic peroxidases especially lignin peroxidase (LiP, E.C.1.11.1.14) and manganese peroxidase (MnP, E.C.1.11.1.13). These fungal peroxidases are all ligninolytic heme-containing glycoprotein enzymes that require H2O2 as an oxidizing agent (Kähkönen et al., 2017).

The fungal tolerance to micropollutants such as polycyclic aromatic hydrocarbons (PAHs) has been used in previous studies as preliminary assessment to identify the promising PAH-degrading fungal strains, for biotechnology applications (Lee et al., 2014). The tolerance to the micropollutants has been reported to be related to the ligninolytic enzymes production in *Basidiomycetes* fungi, involved in the oxidation of wide range of pollutant compounds with aromatic structures, including polycyclic aromatic hydrocarbons (PAHs), PhCs and synthetic dyes (Lee et al., 2014). According to Andreolli et al. (2016) fungal strains which exhibit a tolerance above 70% could be useful for biodegradation of PAHs and organic xenobiotic compounds in wastewater. The aim of this study was to assess the simultaneous removal of carbamazepine (CBZ), diclofenac (DCF), and ibuprofen (IBP) by previously isolated fungi (*Aspergillus niger, Mucor circinelloides*, *Trichoderma longibrachiatum*, *Trametes polyzona* and *Rhizopus microsporus*) in liquid media.

## Materials and Methods

### Preparation of Pharmaceutical Compound Solutions

All three selected PhC standards were of high purity grade purchased from Sigma Aldrich, South Africa: carbamazepine (CBZ, 99.7%), diclofenac sodium (DCF, 99.8%), and ibuprofen (IBP, 98%). Both individual standards and combined stock solutions (100 mg/L) were prepared in 100 mL volumetric flasks by dissolving 10 mg of CBZ, 10 mg of DCF and 10.2 mg of IBP separately in 10 mL of methanol (HPLC grade, Sigma Aldrich, South Africa) prior to topping up to the mark with double de-ionized water (ddH2O). The drug stock solutions were wrapped with aluminum foil and stored without light at 4°C. The stock solutions were also renewed on a weekly basis. A mixed solution of three PhCs was also prepared using appropriate dilution of individual stock solutions in ddH2O. The working standard solutions were prepared by spiking an appropriate drug volume of the stock solution in the autoclaved media or in the aerated batch flasks of liquid media as described below.

### Preparation of the Fungal Cultures and Inocula

Five grams of wet pallets of isolated fungi (*A. niger* [OL311502], *M. circinelloides* [OL311501], *R. microsporus* [OL311505], *T. longibrachiatum* [OL311503] and *T. polyzona* [OL311504]) each were used for the purpose of this study. Prior to mycelium growth, previously identified fungal isolates were plated on solid medium consisted of D-(+)-glucose anhydrous (40 g/L, Sigma Aldrich, South Africa), peptone (10 g/L, Sigma Aldrich, South Africa) and agar (15 g/L, Sigma Aldrich, South Africa). After 5 days of incubation at 30 ± 1.5°C, mycelial pellets (approximately 5.9 ± 0.1 g in dry weight) were harvested from the solid media, added in 100 mL of liquid low nitrogen medium (LN-m). The mixture was homogenized and aliquots with fungal suspensions were used as inoculum for the rest of the experiments. The pellets were measured as wet weight and later converted to dry weight by the ratio wet weight/dry weight measured for each fungal isolate as reported by Vasiliadou et al. (2016). The LN-m has been used since it showed good growth of the selected fungal isolates as described by Kasonga et al. (2021). The LN-m media consisted of 100 mL of Basal III medium, 100 mL of 0.1 M of trans aconitic acid (pH 4.3), 0.2 g of ammonium tartrate bibasic and 60 mL of trace element solution. The trace element solution contained 1.5 g of Nitrilotriacetic acid pH 6.5 (pH adjusted using NaOH or HCl, 1M), 0.5 g of MnSO_4_.H_2_O, 1g of NaCl, 0.1 g of FeSO_4_.7H_2_O, 0.1 g of CoCl_2_.6H_2_O, 0.1 g of ZnSO_4_.7H_2_O, 0.1 g of CuSO_4_.5H_2_O, 10 mg of Al_2_(SO_4_)_3_.18H_2_O, 10 mg of H_3_BO_3_ and 10mg of Na_2_MoO_4_.2H_2_O per liter of solution. The media was also supplemented with 1 mL of sterile thiamine chloride solution (1mg/mL) and 10 g/L of D-(+)-glucose anhydrous as vitamin and carbon sources, respectively.

### Assessment of the Fungal Tolerance to Pharmaceutical Compounds

The tolerance index of the five fungal strains (*T. longibrachiatum, T. polyzona, A. niger, M. circinelloides* and *R. microsporus*) was in a combination of the three selected PhCs (CBZ, DCF, and IBP) at different concentrations in solid medium and in liquid media (200mL aerated test flasks).

#### The Fungal Tolerance to Pharmaceutical Compounds in Solid Medium

Prior to their addition aseptically to the autoclaved solid medium, the standards for the selected PhCs (CBZ, DCF, and IBP, Sigma Aldrich, South Africa) were prepared by dissolving separately in HPLC grade methanol (Sigma Aldrich, South Africa) then made up to the mark in 100 mL volumetric flask of stock solution (100 mg/L) with ddH2O. The basic test medium contained: D-(+)-glucose anhydrous (40 g/L), agar (15 g/L) and peptone (10 g/L) (named medium D). Medium D for indicator plates were supplemented with CBZ, DCF, and IBP individually and in a combination using the following concentrations: 0.1, 1, 5, 10, and 15 mg/L of each drug. The plates were inoculated with ± 4 mm of fungal mycelium disk and incubated at 30 ± 1.5°C for 7 days. The drug’s fungal tolerance index test was run in triplicate and calculated by evaluating the fungal mycelial mat according to the following Equation (1) as described by previous investigators (Lee et al., 2014; Andreolli et al., 2016).


(1) FTR=(DFT/DFC)×100

Where: FTR (%) is the fungal tolerance; DFT (cm) is the diameter of fungal growth in test plate and DFC (cm) represents the diameter of fungal growth in control plate. Fungal growth diameters were evaluated by measuring the expanding colonies (Ravimannan et al., 2014; Reyes-César et al., 2014; Sardar et al., 2016).

#### The Fungal Tolerance to Pharmaceutical Compounds in Liquid Media

The experiment was run in triplicate (*n* = 3) in 500 mL aerated batch flasks, using a mixture which contained all three drugs in 200 mL of liquid media. The aerated batch flasks contained 10 g/l of D-(+)-glucose anhydrous, 0.2 g/L of ammonium tartrate dibasic and 1.5 mL of sterile 1% Tween^®^ 80 solution (MINEMA Chemicals, South Africa) (Tien and Kirk, 1988). The three selected PhCs were assessed, at room temperature, using the following concentrations: 1, 2.5, 5, 7.5, and 10 mg/L. The flasks were inoculated with 10 and 30% of the volume of the mycelium suspension and incubated at 30 ± 1.5°C. The use of 30 ± 1.5°C as optimum temperature was according to Kasonga et al. (2021) who reported the suitable temperature for fungal growth ranging from 25 to 35°C with almost an optimum at 30 ± 1.5°C.

The experiments were run for 9 days, after which fungal biomass formation was evaluated. The FTR was calculated using Equation (1) by considering DFT (mg/200mL) as the fungal biomass in the test batch supplemented with drugs, whereas DFC (mg/200mL) was the fungal biomass in the control batch. Determination of the fungal biomass from the aerated batch was carried out by weighing the filtered dry biomass (Patel et al., 2017). A tolerance index larger than 70% (in solid or suspension liquid media) was demonstrated to be promising of *in vitro* fungal bioremediation (Andreolli et al., 2016).

### Enzymatic Activity Assays

#### Screening of Ligninolytic Enzymes Activity in Solid Medium

As described by previous investigators (Kähkönen et al., 2017), the basic test solid medium contained per liter the following compounds: 10 g of D-(+)-glucose anhydrous, 0,5 g of MgSO4.7 H2O, 2 g of KH2PO4, 0,1 g of CaCl2, 0,5 g of ammonium tartrate, 0,1 g of yeast extract and 25 g of agar. A solution of 2,2 dimethyl succinate (Sigma Aldrich Ltd., China) was used as buffer (pH 5.0) and the pH of the medium was adjusted using 1M NaOH or 1M HCl, before autoclaving. One hundred milliliter (100 mL) of this medium was aseptically transferred to separate 250 mL sterile Erlenmeyer flasks, which were supplemented with mediator substrates for the enzymatic activity assay as described by Kähkönen et al. (2017) with some modifications. Briefly, the medium was amended with 25 mg/kg of ABTS (Sigma Aldrich, South Africa) prepared in 25 mM sodium acetate buffer solution (pH 4.5) for Lac assay (Collins et al., 1998).

The final medium was aseptically transferred to plates, which thereafter, were inoculated with ± 4 mm diameter of fungal mycelium from pre-cultured medium D. At least three plates were inoculated with individual isolated fungal strains and incubated at 30 ± 1.5°C. The control plates (three for each fungal strain) without the mediator substrates for enzymatic activity assay were also inoculated with the fungal mycelium and incubated at 30 ± 1.5°C. All the plates were incubated for 7 days and the growth was allowed to extend for 14 days in order to record any specific color production. A distinctive color zone produced by fungi growing in indicator plates (with mediator substrate) was able to indicate the presence of the targeted enzyme in solid medium.

Although the method mentioned above was especially described for Lac, in the present study, it was also extended for the detection of other enzymes (MnP and LiP) by using appropriate mediator substrates. The solid medium was supplemented with 25, 10, and 1 mL/L of VA (96%, Sigma-Aldrich, South Africa) for the LiP activity assay (Tien and Kirk, 1988; Silva Lisboa et al., 2017). The VA solution of 20 mM was prepared in 0.2 M sodium tartrate buffer (pH 3.0). The medium was also supplemented with 25 mL/L of 2,6-DMP (Sigma-Aldrich, Chemie GmbH, South Africa) for the MnP activity assay (Martinez et al., 1996; Amriani et al., 2017). A solution of 2,6-DMP at 1 mM was prepared in 0.5 M sodium tartrate buffer (pH 4.5). The fungal colonies were observed after 7 and 14 days of incubation for colored zones around and below the surface of their colony in the substrate containing plates.

#### Assessment of Ligninolytic Enzyme Activity in Liquid Media

Preparation of crude enzymes – the samples were collected from the 250 mL Erlenmeyer flasks containing 100 mL of 5-days-old fungal mycelium solution. The crude supernatant LN-m was filtered (Whatman^®^ Mixed Cellulose, 0.45 um) to remove the mycelium and spores (Lee et al., 2014). Assessing the enzymatic activity assay – the ligninolytic enzymatic activity assay of the indigenous isolated fungal strains was carried out using a UV-Vis Spectrophotometer (HACH model DR 6000™ CO, United States, provided with 10 mm glass cells) at room temperature. Experiments were performed in triplicate using fresh samples of 5-days-old culture in LN-m prepared as described by Tien and Kirk (1988). Lac activity was assessed through the oxidation of 5 mM ABTS in 100 mM sodium acetate buffer (pH 5.0) at 420 nm. The increase in absorbance of the substrate ABTS at 420 nm was followed at the extinction coefficient (ε) of ε420nm = 3.6 × 10^4^ M^–1^cm^–1^ (Collins et al., 1998; Ryan et al., 2005). The LiP activity was determined by monitoring the oxidation of 20 mM VA in 0.2 M sodium tartrate buffer (pH 3.0) with 2 mM hydrogen peroxide at 310 nm. The extinction coefficient considered was ε310 nm = 9.3 × 10^3^M^–1^ cm^–1^ to monitor the increase in substrate absorbance (Tien and Kirk, 1988; Silva Lisboa et al., 2017). The MnP activity was determined by monitoring the oxidation of 20 mM 2,6-DMP in 0.5 M sodium tartrate buffer (pH 4.5) and 20 mM manganese sulfate with 2 mM hydrogen peroxide at 470 nm. The extinction coefficient ε470 nm was 49.6 × 10^3^M^–1^cm^–1^ (Martinez et al., 1996; Amriani et al., 2017). In addition, the fungal enzymatic activities were expressed as international units (U), which are defined as the enzymatic amount required to transform or oxidize 1 μmol of substrate per minute (Ryan et al., 2005). The enzymatic activities evaluated in U/mL were thereafter expressed in U/L in the results. Effect of pH on ligninolytic enzymatic activity – fungal cultures in LN-m were incubated for 5 days at 30 ± 1.5°C using different initial pH values ranging between 2.3 and 6.2 (means 2.3, 3.1, 4.31, 5.5, and 6.2) to establish the influence of initial pH on enzyme production in LN-m as described above. Effect of temperature on ligninolytic enzymatic activity – the optimum temperature for enzyme production was determined by incubating, in triplicate fungal cultures in LN-m medium (pH 4.3) for 5 days at five (5) different temperatures (20 ± 1.5, 25 ± 1, 30 ± 1.5, 37 ± 1, and 45 ± 1°C). The enzymatic activities were assayed as described above. Effect of incubation time on ligninolytic enzymatic activity – the effect of incubation time on enzyme production was evaluated between 3 and 15 days as described above, in LN-m medium (pH 4.3 at 30 ± 1.5°C). The fungal enzymatic activity was therefore assayed for 3, 6, 9, 12, and 15 day old cultures.

### Experimental Design for Pharmaceutical Compounds Degradation

The biodegradation experiments of the three (3) targeted PhCs, namely CBZ, DCF, and IBP, by the isolated fungal strains, were conducted at room temperature, in continuously aerated 500 mL glass flasks containing a working volume of 200 mL of liquid media. The composition of the liquid media in the test flasks was made. The control batch flask comprised the liquid media with 30% of LN-m without isolated fungi. The CBZ, DCF, and IBP were spiked at a concentration of 1 mg/L of each. The rationale to use this relatively high concentration range was the possible application of the fungal bioremediation technology in the treatment of drug manufacturing effluents. Reviews about drug manufacturing discharge of active pharmaceutical ingredients (APIs) found concentrations of up to 1 mg/L downstream from these factories (Cardoso et al., 2014; Larsson, 2014).

**FIGURE 1:**
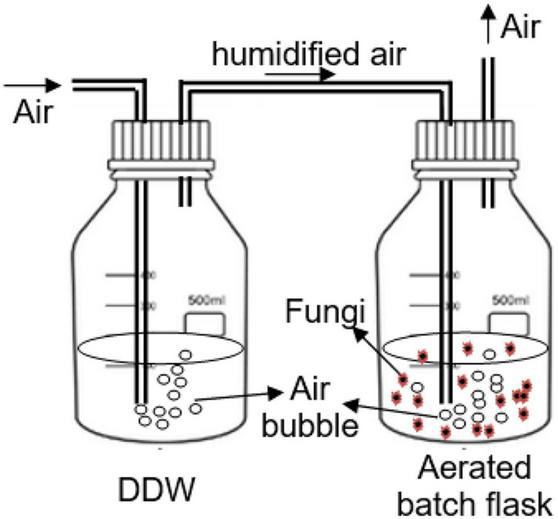
Setup of the continuously aerated batch flasks (ABFs).

Figure 1 shows the setup of the aerated batch flasks (ABF) for the evaluation of the selected PhCs removal by individual isolated South African indigenous fungal strains. The experiments were run in duplicate (*n* = 2) for the period of 14 days and samples were taken daily from the day 0 to 5, then at days 7, 10, and 14. Collected samples (1 mL) were diluted to 10 mL by adding 9 mL of ddH2O at pH 2.5 before loading onto the cartridge column (Supel™-Select Supelco-HLB 500 mg, 12mL).

The pH of each batch flask was adjusted daily to around 4.3 by adding 1M HCl or 1M NaOH to avoid the inactivation of the fungal extracellular enzymes over the pH range of 3.5 to 4.8 (Yang et al., 2013b). Batch flasks were wrapped with aluminum foil to avoid the influence of light on PhCs stability and therefore the removal efficiency by fungi. The air supplied in the test flasks was humidified through distilled water flasks. After bubbling through the test flask, the air was allowed to evacuate throughout a tube. The humidified air was preferred in the aeration laboratory system to minimize solution volume losses by evaporation during the experiment.

In Figure 1, TL was the isolated fungal strain 1 (*T. longibrachiatum)*, TP the isolated strain 2 (*T. polyzona)*, AN the isolated strain 7 (*A. niger)*, MC the isolated strain 8 (*M. circinelloides)* and RM the isolated strain 11 (*R. microsporus)*, while Ctr was the control ABF without fungi.

### Solid-Phase Extraction–Ultra Performance Liquid Chromatography Tandem Mass Spectrometry Methods

All the solvents used for UPLC-MS analysis were ultra-pure grade solvents, ultrapure HPLC grade (Romil-UpS™, Microsep, South Africa). Ultra-pure grade methanol and water used as eluent were spiked with 0.1% formic acid (99%, Thermo Scientific, South Africa).

#### Solid-Phase Extraction Method

Three sorbents cartridges were evaluated for simultaneous extraction of the targeted PhCs from aqueous matrices, namely Supel™-Select Supelco-HLB 60 and 500 mg, 12 mL plastic cartridges (hydrophilic polymers typically comprised of a hydrophobic component such as polystyrene and/or divinylbenzene, and a hydrophilic component including vinylimidazol, methacrylate, N-vinylpyrillidone, and/or hydroxyl, Sigma Aldrich, South Africa), Oasis^®^ HLB 60 μm (LP) 500cc glass cartridges (a co-polymer of vinylpyrrolidone and divinylbenzene, Water Microsep Pty Ltd., South Africa) and Discovery^®^ DSC-18 SPE Tube 500 mg, 6mL plastic cartridges [Si(CH2)17CH3 a polymerically bonded, octadecyl silica, 18% C endcapped, Sigma Aldrich, South Africa]. Their performance was tested by comparison with recoveries at pH 2.5, 3, and 5. The preliminary tests were accomplished by loading in the cartridge column a volume of 10, 100, and 200 mL of PhC at concentrations of 0.1 and 1 mg/L. From these results it was decided to use the pre-conditioned Sorbent Supel™-Select Supelco-HLB (hydrophilic-lipophilic balance) Resin-54184-U, 500 mg/12 mL plastics (Sigma Aldrich, South Africa) cartridges at pH 2.5 (Castiglioni et al., 2005, 2006; Agunbiade and Moodley, 2016; Van Zijl et al., 2017). Prior to the loading of the liquid media samples onto the Solid-Phase Extraction (SPE) column (10 mL each) at the flow rate of ±1 mL/min (drop by drop) for PhCs extraction efficiency, these water samples were filtered through 0.45 μm Whatman filter paper (GR91 with 12.5 cm diameter), at pH 2.5 (adjusted with 1M HCl). Subsequently, the SPE Supel™-Select HLB cartridges were air-dried completely by vacuum for 30 min. The PhCs were eluted with 6mL of pure methanol kept for 30 min. Complete elution of PhCs was performed at the same flow rate (drop by drop). The extracts were collected in 10 mL tube and concentrated at room temperature under a gentle stream of nitrogen using an evaporation unit (Thermo Scientific Reacti-Vap 1, Germany) to bring the sample volume to 1mL, which was transferred to a 1.5 mL amber vial. Extracts were dried completely under nitrogen and were reconstituted to a final volume of 1mL of water- methanol (1:1, v/v) for ultra-performance liquid chromatography-tandem mass spectrometry (UPLC/MS) injection.

#### Ultra-Performance Liquid Chromatography-Tandem Mass Spectrometry Method

The PhCs separation and their detection were performed using a Waters^®^ Synapt G2 high definition mass spectrometry (HDMS) system (Waters Inc., Milford, MA, United States). The system comprises of a Waters Acquity Ultra Performance Liquid Chromatography (UPLC®) system hyphenated to a quadrupole-time-of-flight (QToF) instrument. The system operated with MassLynx™ (version 4.1) software (Waters Inc., Milford, MA, United States) for data acquisition and processing. An internal lock mass control standard of 2 pg/μL leucine enkephalin solution (*m/z* 555.2693), was directly infused into the source through a secondary orthogonal electrospray ionization (ESI) probe, which allowed the intermittent sampling. The internal control was used to compensate for instrumental drift, ensuring relevant mass accuracy, throughout the duration of the runs. The instrument was calibrated using sodium formate clusters and IntelliStart functionality (mass range 112.936–1 132.688 Da). Resolution of 20 000 at *m/z* 200 [full width at half maximum (FWHM)] and mass error within 5 mDa were obtained. The source conditions were as follows: the capillary voltage for ESI of 2.6 kV for positive mode ionization, the source temperature was set at 120 oC, the sampling cone voltage at 25 V, extraction cone voltage at 4.0 V and the cone gas (nitrogen) flow at 10.0 L/h. It is important to mention that the desolvation temperature was set at 350 oC with a gas (nitrogen) flow of 600.0 L/h. Quantitative data-independent acquisition (DIA) was done using two simultaneous acquisition functions with low and high collision energy (MS*^E^* approach) with a QTOF instrument. Fragmentation was performed using high energy collision induced dissociation (CID). The fragmentation energy was set at 2 and 3 V for the trap and collision energy, respectively. The ramping was set from 3 to 4 and 20 to 40 V for the trap and transfer collision energy, respectively.

The mass spectral scans were collected at a frequency of every 0.3 s. The raw data were collected in the form of a continuous profile. Mass to charge ratios (*m/z*) between 50 and 1 200 Da were recorded. Separation was completed using a reverse phase step gradient elution scheme from 60% H2O (0.1% formic acid) to 95% methanol (0.1% formic acid). The gradient started with an isocratic flow (hold 0.5 min) followed by a linear increase to 95% methanol (6.5 min); subsequently, the column was washed for 0.8 min followed by conditioning and re-establishment of initial conditions to allow for equilibration before the start of the next run for the complete elution scheme. The column temperature was kept constant at 40 oC and the flow rate was set at 0.3 mL/min for the entire run giving a total run time of 9 min. Injection volumes were set at 5 μL and Column: Titan C18 80 Å(2.1 mm ID × 100 mm length, 1.9 μm particle size) (Supelco, Sigma-Aldrich Ltd., South Africa) was used to perform the analysis.

#### Method Development and Validation

A control blank matrix extract with increments of known analyte concentrations of selected drugs (CBZ, DCF, and IBP) was used for the method development. The blank matrix (ddH2O) was spiked with a mixture of PhC standard solutions (CBZ, DCF, and IBP) at the concentration of 0.01, 1, and 5 mg/L. According to their monoisotopic mass, all targeted drugs were quantified in positive ion mode (ESI+) while their detection in low energy was performed in a reaction monitoring mode of, respectively, *m/z* 237.10 (CBZ), *m/z* 296.02 (DCF), and *m/z* 229.12 (IBP for IBP+Na). The following Table 1 shows the method development parameters set to perform the UPLC/MS.

**TABLE 1:** UPLC/MS method development parameters of selected pharmaceuticals.

Analytes	Therapeutic class	m/z	Rt/min	Linear-range mg/L	Noise start	Noise end
CBZ	anti-epileptic	237.10	2.750 ± 0.1	0.001–2	2.5	2.6
DCF	anti-analgesic	296.02	4.903 ± 0.2	0.001–2	4.1	4.5
IBP	anti-analgesic	229.12	4.995 ± 0.3	0.001–2	4.5	4.6

Considering the endogenous compounds from the aerated batch flasks (including fungal enzymes and other compounds), the ionization of the targeted analytes could be increased or suppressed. The effect of the fungal medium constituents as a matrix effect was evaluated by comparing the slope of the selected PhC calibration curves in spiked medium extracts and in spiked pure solvent (Zhao et al., 2018). The matrix effect using the extracted SPE HLB column was evaluated and the values less than 20% were considered acceptable (Zhao et al., 2014). Equation (2) was used to determine the matrix effect (*ME*) in the working conditions as follows:


(2) $$ME=\frac{S\,l\,o\,p\,e\,o\,f\,f\,u\,n\,g\,a\,l\,m\,e\,d\,i\,u\,m-m\,a\,t\,c\,h\,e\,d\,c\,a\,l\,i\,b\,r\,a\,t\,i\,o\,n\,c\,u\,r\,v\,e}{S\,l\,o\,p\,e\,o\,f\,t\,h\,e\,s\,t\,a\,n\,d\,a\,r\,d\,c\,a\,l\,i\,b\,r\,a\,t\,i\,o\,n\,c\,u\,r\,v\,e\,e}\times \,100$$

The method was thereafter validated in terms of the required parameters including: linearity of the calibration curve, specificity and selectivity, sensitivity, precision and accuracy, and recovery (Lee et al., 2008; Kasonga et al., 2019a,b).

##### Linearity of Calibration Curve

Calibration curves were carried out by plotting the peak area ratios (or signal response) of each analyte versus the theoretical eight (8) point concentrations of the spiked drug in methanol-water (1:1, v/v). The mixture CBZ, DCF, and IBP at individual concentrations of 0.001, 0.01, 0.1, 0.25, 0.5, 1, 1.5, and 2 mg/L was prepared in triplicate for each concentration from filtered fungal batch media. The filtered medium without selected PhCs was used as a blank. Prior to the injection in UPLC/MS, the SPE was performed for all blank and analyte media. The linearity of the calibration curves was assessed throughout the recorded *r*^2^ values and equations given by the instrument. The recorded values were found to be in the acceptable range >0.9 as suggested in literature (Wang et al., 2011; Wabuyele, 2013).

##### Sensitivity

The sensitivity of the method was evaluated in terms of the limit of detection (LoD) and the limit of quantification (LoQ) for each selected PhC and was assessed during the determination of the linear range of the calibration standards as those concentrations giving a signal to noise ratio (S/N) of 3 and 10, respectively. The LoD and the LoQ were calculated according to Gracia-Lor et al. (2010).

##### Accuracy and Precision

To assess the reproducibility and measuring the closeness among the replicates, daily precision and accuracy, CBZ, DCF, and IBP (mixed working standard solution) were considered as follows: at low (0.01 and 0.1 mg/mL), middle (0.75 and 1 mg/L), and high (2 mg/L) concentrations. These concentrations were spiked in triplicate (*n* = 3) in accordance with the method proposed by previous investigators (Gracia-Lor et al., 2010; Ananthula et al., 2015). The daily precision (intra-day/inter-day assay), one spike per day over 3 days, was then assessed by determining the method accuracy (expressed as percent recovery) and precision [expressed as repeatability in terms of percent relative standard deviation (%RSD)]. The criteria for tolerability were that the precision level should not exceed 15–20% and the averaged assessment of the accuracy should be within ±15–20% (Wang *et al.*, 2011).

##### Specificity and Selectivity

These parameters were evaluated in terms of chromatographic interferences from endogenous compounds which were eliminated by comparing the chromatograms of the blank medium and the analyte spiked medium (CBZ, DCF, and IBP) as described by previous investigators (Wabuyele, 2013; Ananthula et al., 2015). The analyte samples at the concentrations of 0.001, 1, and 5 mg/L were used for the study.

Identification of the known metabolites of selected PhCs–Full-scan MS data collected on a QToF-MS instrument under ESI^+^ conditions for the samples from day 3 and day 10 of each aerated batch flask were followed by acquisition of the product ion spectrum. Therefore, protonated molecules and sodium adduct products at accurate *m/z* were tentatively assigned to identified metabolites of the selected PhCs (CBZ, DCF, and IBP).

### Statistical Analysis

The analysis of data was carried out using R software version 3.4.1 (Oksanen et al., 2013). Data were expressed in this study as means ± standard deviations, in order to run a one-way ANOVA. The level of significance of *p* < 0.05 for F test was considered to establish significant differences among the means.

## Results

### Fungal Tolerance Study

**FIGURE 2:**
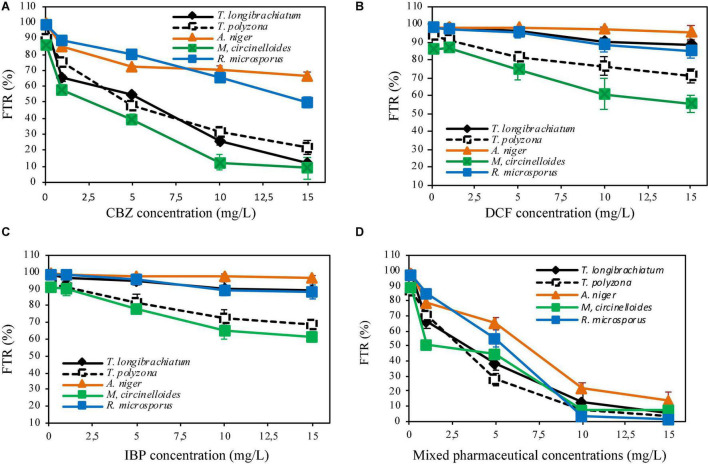
Fungal tolerance to CBZ **(A)**, DCF **(B)**, IBP **(C)** and mixed pharmaceuticals **(D)** in solid medium.

**FIGURE 3:**
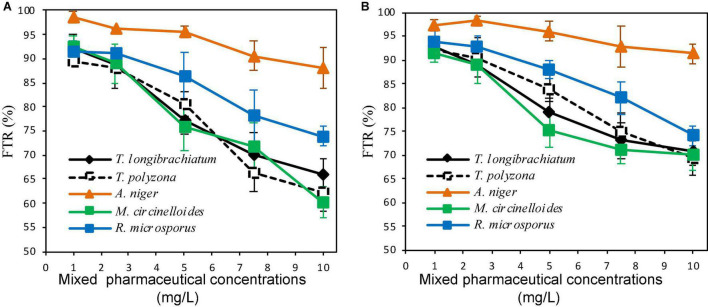
Fungal tolerance in liquid media of mixed PhC concentrations of CBZ, DCF, and IBP (**A,B** are aerated batch flasks with 10 and 30% of fungal inoculum, respectively).

The fungal tolerance index to PhCs in solid media or suspended in liquid media performed in indicator plates or in aerated batch flasks (ABFs) are shown in Figures 2, 3. It revealed a gradual decrease in fungal mycelial mat (in indicator plates) or in fungal biomass (dry mass, in ABFs) with an increase in PhC concentrations. A tolerance index of over 70% could be promising of the fungal biodegradation of the selected PhCs.

#### Fungal Tolerance to Pharmaceutical Compounds in Solid Medium

The investigation of the level of tolerance of the five (5) isolated indigenous fungi to targeted PhCs in solid medium showed that, all fungal strains (100%), demonstrated in general the tolerance to the selected PhCs at different concentrations. A high tolerance index of more than 80% was recorded for all the fungi when individual drug were spiked at the concentrations of 0.1 mg/L for CBZ (Figure 2A) and at 0.1 to 5 mg/L for DCF and IBP (Figures 2B,C). With a mixture of three drugs spiked at the concentration of 0.1 mg/L of each, a tolerance index of over 80% was also recorded.

In indicator plates amended with CBZ at a concentration of 1 mg/L, *A. niger, R. microsporus* and *T. polyzona* exhibited a tolerance index of over 70% (85.11 ± 1.01, 88.62 ± 0.21, and 75.21 ± 1.01%, respectively), while the tolerance index of *M. circinelloides* and *T. longibrachiatum* were found to be below 70% (58.11 ± 2.55 and 65.12 ± 2.01%, respectively) (Figure 2A). At CBZ concentration of 5 mg/L, *A. niger* and *R. microsporus* displayed a tolerance index of 72.15 ± 0.88 and 80.12 ± 2.11%, and only *A. niger* tolerated CBZ concentration of 10 mg/L with FTR value of 70.12 ± 2.65%. The PhCs (DCF and IBP) were found to be tolerated by all fungal strains (FTR > 70%) up to the concentration of 15 mg/L, except by *M. circinelloides*, which could tolerate the concentrations up to 5 mg/L [74.11 ± 5.11% for DCF (Figure 2B) and 78.09 ± 1.99% for IBP (Figure 2C)]. However, only *A. niger, T. polyzona* and *R. microsporus* demonstrated the tolerance index to selected PhCs up to 1 mg/L in plates supplemented with mixture PhCs (FTR > 70%, Figure 2D).

No fungal strain was found to tolerate PhCs at concentration over 5 mg/L. *M. circinelloides, T. longibrachiatum* and *T. polyzona* strains were significantly affected (*p* < 0.05) by the increasing CBZ concentration to over 1 mg/L, compared to *A. niger* and *R. microsporus*. Amongst the fungal strains, *M. circinelloides* was the most significantly affected in drug supplemented plates amongst the isolated strains. A significant reduction in the fungal tolerance index (*p* < 0.05) was observed for *M. circinelloides* from the concentration of 1 mg/L, while *T. longibrachiatum* could tolerate this concentration.

#### Fungal Tolerance to Pharmaceutical Compounds Suspended in Liquid Media

In the ABFs inoculated with 10% of fungal mycelium solution, a tolerance index > 70% was recorded for PhC mixture concentrations up to 7.5 mg/L for all fungal strains, except for *T. polyzona* (66.28 ± 3.99%). Above this concentration, only *A. niger* and *R. microsporus* were found to be able to tolerate PhC mixture concentration of 10 mg/L (87.99 ± 4.31 and 73.98 ± 2.12%, respectively) (Figure 3A). When 30% of fungal inoculum was used, all fungal strains were able to tolerate (FTR > 70%) the PhC concentrations up to 10 mg/L (Figure 3B). However, regardless of fungal inoculum concentration, the decrease in fungal biomass was substantial from PhC concentrations of 3 mg/L, 5 mg/L and above, except for *A. niger*. For this strain, the biomass decrease appeared not to be significant (*p* > 0.05). Regardless of fungal species, the batch inoculated with 30% demonstrated a high tolerance index compared to the fungal inoculum concentration of 10%.

As can be seen in Figure 3, the lowest FTR (%) of 69.29 ± 3.69% was recorded with *T. polyzona*, while 60.33 ± 3.23% was observed with *M. circinelloides* when 10% fungal inoculum was used. *Aspergillus niger* showed a higher PhC tolerance index regardless of the fungal inoculum concentration in batch flasks, followed by *R. microsporus.* The FTR (%) values recorded at various PhC concentrations for *T. longibrachiatum, T. polyzona* and for *M. circinelloides* were found to be in the same range (*p* > 0.05).

### Assessment of the Simultaneous Degradation of Carbamazepine, Diclofenac, and Ibuprofen by the Isolated Fungi

#### Method Development and Validation

##### Solid Phase Extraction

The finding of the appropriate sorbent and extraction conditions that lead to acceptable recoveries for the three targeted PhCs CBZ, DCF, and IBP has been evaluated. The widely used cartridges for PhCs extraction namely HLB [Oasis^®^ HLB (a co-polymer of vinylpyrrolidone and divinylbenzene) and Supel™-Select Supelco HLB (a hydrophilic polymers typically comprised of a hydrophobic component such as polystyrene and/or divinyl benzene, and a hydrophilic component including vinylamidazol, methacrylate, N-vinyl pyrillidone, and/or hydroxyl)] and to Discovery^®^ DSC-18 [Si(CH_2_)_17_CH_3_ a polymerically bonded, octadecyl silica, 18% C endcapped] were compared. Regardless of the volume loaded, recoveries of the three sorbents column at tested conditions were as followed: the Supelco-Select HLB and Oasis HLB at pH 2.5 and 3 yielded over 90% recoveries for the three selected analytes (98 ± 7%), with slight increase in recovery at pH 2.5. The Supelco HLB cartridge containing 500 mg sorbent were found recovering the compounds significantly (*p* < 0.05) better than the cartridge containing 60 mg sorbent. In the contrary, at pH 2.5, 3, and 5, recoveries lower than 80% (75 ± 4.6%) were recorded for the Discovery DSC-18 cartridges. Therefore, recovery results indicate that both HLB cartridges (Oasis and Supelco-Select) were found to be suitable for the simultaneous extraction of the three selected PhCs CBZ, DCF, and IBP. However, the Supelco-Select HLB cartridges were taken because of their low cost, knowing that large number of samples were to be processed. The lower pH of 2.5 was selected in order to optimize the analytes extraction, as the pH appeared to affect recoveries.

##### Ultra-Performance Liquid Chromatography-Tandem Mass Spectrometry Analysis

**FIGURE 4:**
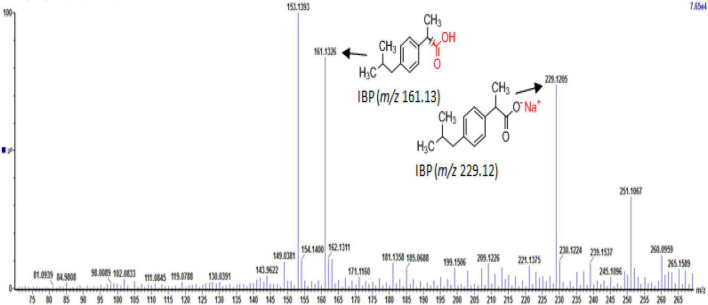
UPLC-(+)-ESI-QToF-MS production spectra of IBP analyte at m/z 161.13 and 229.12.

Full-scan and product ion scan were performed in order to optimize the MS conditions of targeted analytes in ESI^+^ and ESI^–^ mode, respectively. MS mass spectra of selected PhCs were obtained from injection of 1 mL of 0.001, 1, and 5 mg/L methanol/water (1:1, v/v) individually and combined in standard solutions. The three selected compounds were analyzed under positive ionized mode and low energy, which conditions were found to be highly efficient. The selective UPLC/MS chromatograms and spectrum of targeted analytes CBZ (Supplementary Figure 5A, *m/z* 237.10), DCF (Supplementary Figure 5B, *m/z* 296.02) and IBP (Supplementary Figure 5C, *m/z* 161.13) with their monoisotopic mass, respectively, were selected as parent ions. IBP gave two compounds fragmented with intense product ion signal at *m/z* 161.13, when considering the elimination of fragment −CHO_2_ from IBP [M − CHO_2_], and fragment [M+Na^+^] at *m/z* 229.12 (Supplementary Figure 5C and Figure 4). However, IBP quantification was performed with the second fragment selected as parent ion. Table 2 above summarized the optimized main spectrometric parameters for achieving the maximum abundances of PhCs and fragment ion presents in the working conditions. Better resolution of PhC’s peaks was achieved with water/methanol mixture. The 0.1% formic acid in the mobile phase contributed to obtain the smallest peak of analytes. Therefore, a linear gradient program of the SPE-UPLC/MS analysis resulted in better peak symmetric and separation as demonstrated in Supplementary Figures 5A–C.

**TABLE 2:** Method validation parameters.

*Analytes*	*Experimental r^2^*	*Calculated r^2^*	*LoD (mg/L)*	*LoQ (mg/L)*	*Mean recovery (%)*	*RSD (%)*
CBZ	0.969	0.980	9.71 × 10^–5^	3.24 × 10^–4^	106.20	8.45
DCF	0.965	0.985	2.4 × 10^–4^	8.1 × 10^–4^	104.62	7.33
IBP	0.927	0.957	4.47 × 10^–3^	17.16 × 10^–3^	102.89	8.50

##### Matrix Effect

The matrix effect was found to be negligible after performing the sample purification procedure for the SPE method to reduce the matrix interferences. The matrix factor value (*ME ≈* 0.15%) was less than 20%. Therefore, the signal enhancement or suppression by the matrix effect was found to be non-existent, because of the selectivity of the procedure and Sorbent Supel™-Select Supelco-HLB cartridges used followed by UPLC/MS for the selected PhCs. Hence; none interferences with analytes peaks on their respective retention times were found in the working conditions.

##### Selectivity

**FIGURE 5:**
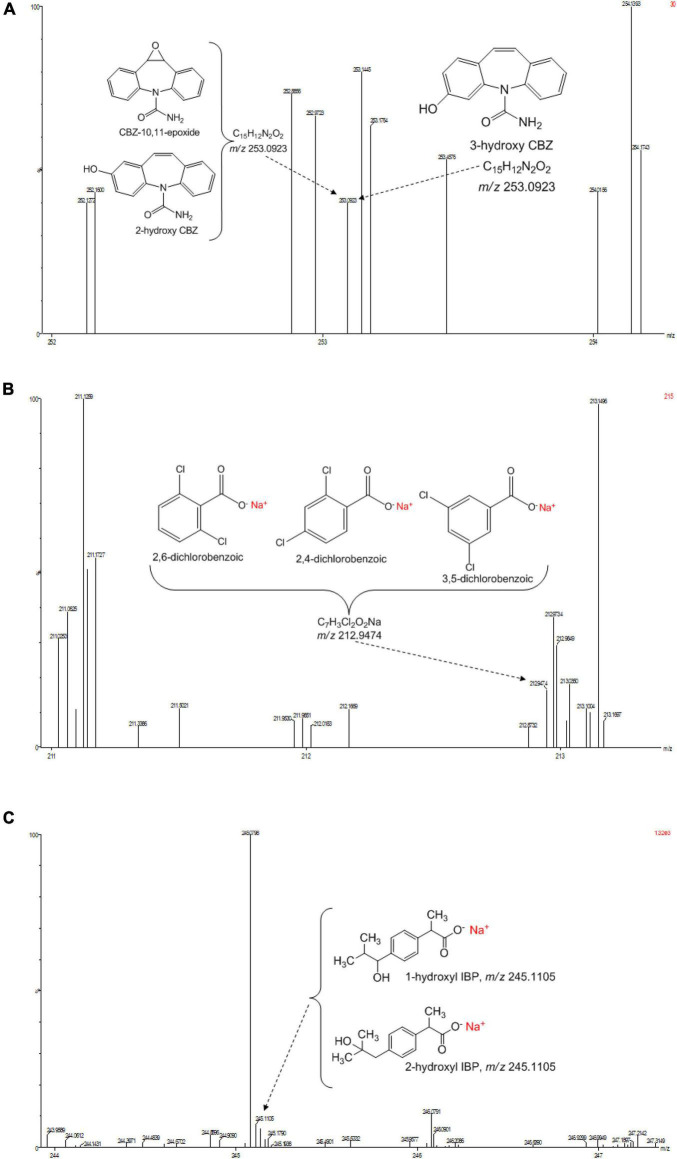
**(A)** Selected UPLC-(+)-ESI-QtoF-MS spectrum of the suggested analyte ion fragments from CBZ. **(B)** Selected UPLC-(+)-ESI-QtoF-MS spectrum of the suggested analyte ion fragments from DCF. **(C)** Selected UPLC-(+)-ESI-QtoF-MS spectrum of the suggested analyte ion fragments from IBP.

The UPLC/MS method selectivity was examined by preparing and analyzing analytes at different the concentrations. From the Supplementary Figure 5, no endogenous peaks were observed at the retention times of each compound given in Table 2. *Linearity and sensitivity:* The good linearity was exhibited by the eight points of the calibration curves for the selected PhCs in the concentration range of 0.001–2 mg/L. The linear regression equations of the PhC calibration curves were observed. The correlation coefficients by linear curves of CBZ, DCF, and IBP were greater than 0.95 as demonstrated in Figures 5A–C as well as in Table 2.

##### Precision and Accuracy

The precision and accuracy of the method were estimated from recovery experiments and RSD of selected PhCs at different concentrations. The closely to 100% recovery values and the RSD values <20% (Table 2) have demonstrated the reliability of the method. The inter-day/intra-day assay for both precision and accuracy were found within acceptable range <15%. *Recovery:* As reported in Table 2 below, the analyte recovery efficiencies (102–106.20%) showed that the SPE method performed with HLB column was found to be efficient to extract the targeted PhCs with negligible interference of matrix components. These results demonstrate that no co-eluting endogenous components significantly affected the analyte ions in the UPLC/MS analytical method. Accordingly, this is establishing the reliability of the analytical method used as well as its subjection to almost non-existent matrix effect.

**FIGURE 6:**
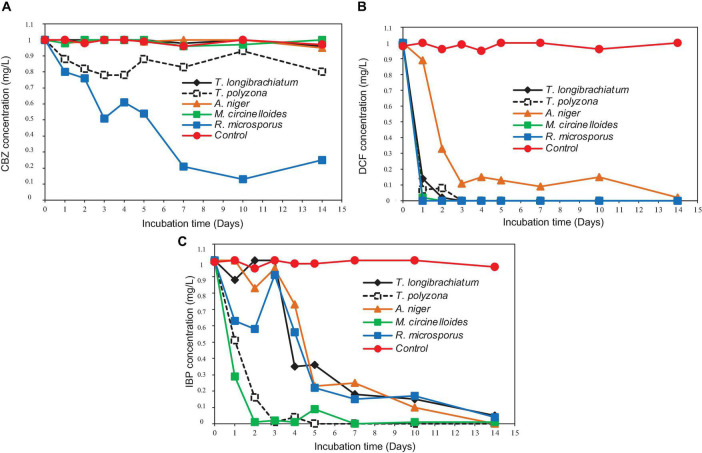
Simultaneous removal of PhCs from liquid media in ABF: **(A)** CBZ; **(B)** DCF; and **(C)** IBP.

Simultaneous removal of PhCs from aerated batch flasks – the following Figures 4, 6A,B display the efficiency of the isolated fungal strains in the simultaneous removal of CBZ, DCF, and IBP by isolated fungal strains in liquid media in the ABF. No PhC removal was observed in the control flasks under the operating conditions. The CBZ seemed not to be removed by *A. niger, M. circinelloides* and by *T. longibrachiatum*, whereas these fungal species have revealed a removal efficiency of over 80% for DCF and IBP. However, CBZ removal of 22% was observed after 3 days with *T. polyzona*, while *R. microsporus* achieved CBZ removal of 20, 24, 49, and 87% within days 1, 2, 3, and 10 (Figure 6A), respectively. In addition, the DCF concentration was found to be below the LoD after day 1 by *R. microsporus*, after day 2 by *M. circinelloides* and after day 3 by *T. longibrachiatum* and *T. polyzona. Aspergillus niger* showed a DCF removal of 11, 67, 89, and 91% at days 1, 2, 3, and 7, respectively. Finally, this fungal strain achieved 98% DCF removal after 14 days (Figure 6B). The Figure 6C depicts a remarkable IBP removal achieved by *M. circinelloides* followed by the WRF *T. polyzona.* From days 1 and 2, the IBP removal of 71 and 99% was recorded for *M. circinelloides*, and 49 and 84% for *T. polyzona. Rhizopus microsporus, A. niger* and *T. longibrachiatum* were able to remove only 42, 17, and 0% of IBP after day 2, respectively. On day 4 and 14, a drastic IBP removal efficiency was observed for *M. circinelloides* (99 and 100%) followed by *T. polyzona* (96 and 100%) and *T. longibrachiatum* (65 and 95%), *R. microsporus* (44 and 96%) and *A. niger* (27 and 100%). From the third day of the experiment, a significant CBZ removal (*p* < 0.05) was observed with *R. microsporus* compared to the rest of the fungi, whereas no improvement was observed in its IBP removal. Although *M. circinelloides* and *T. polyzona* did not perform well in the CBZ removal, they showed a significant IBP removal (*p* < 0.05) compared to the other fungal strains. A better DCF removal was observed for all the fungal strains with lower removal observed for *A. niger* under the operating conditions.

In order to maintain fungal enzymatic activity, pH in the bioreactor was adjusted. Adjusted pH of aerated fungal batch flasks during the experiment – Supplementary Figure 4 shows the pH of the liquid media during the experimental period. During the collection of the aliquots, the pH of this liquid media tended to decrease from the first to fourth day in all the aerated flasks. To maintain it within the optimum range for fungal enzyme production, it was adjusted daily to around 4.3 with the solution of 1M NaOH. The decrease in the pH of the liquid media might be due to the metabolites released by fungi. This decrease in pH continued to be observed in liquid media containing *M. circinelloides* and *T. longibrachiatum* until day 7, while it was stabilized around pH 4.3 in the liquid media with *T. polyzona*. As to the liquid media containing *A. niger* and *R. microsporus*, the pH tended to increase beyond 4.3. Therefore, after 7 days the solution of 1M HCl was used to decrease the pH especially in liquid media containing *A. niger, M. circinelloides*, *R. microsporus* and *T. polyzona* as it tended to increase, except for the liquid media containing *T. longibrachiatum.*

Identification of the CBZ, DCF, and IBP metabolites – samples from day three (3) and day ten (10) of each ABF were collected because it was apparent that better PhC removal could be achieved. Table 3 summarizes accurate mass measurements attributed to molecular fragment ions of the parent compounds and suggested intermediate metabolites from CBZ, DCF, and IBP determined by UPLC-(+)-ESI-QToF- MS. Only few fragment ions and molecular formulas were proposed to correspond to: CBZ (C15H12N2O at *m/z* 237.1095) and its sodium adduct (C15H11N2ONa at *m/z* 259.0814). These were identified in all batch flasks (including the control), which were characterized by high peaks, and small peaks of CBZ-10,11-epoxide, 2-hydroxy-CBZ and 3-hydroxy-CBZ. All of them were found to have a similar empirical formula (C15H12N2O2 at *m/z* 253.0923, Figure 5A), 10,11-dihydro-10,11-dihydroxy-CBZ (C15H13N2O2Na at *m/z* 293.0931), acridine (C13H9N at *m/z* 180.0892), 9-hydroxy- acridine and acridone (C13H9NO at *m/z* 196.0795) from CBZ.

**TABLE 3:** Accurate mass measurements attributed to molecular ions of the parent compounds and intermediates from CBZ, DCF, and IBP determined by UPLC-(+)-ESI-QT oF-MS.

Parent	Compound	Measured mass	Suggested empiric formula	Structure
CBZ	CBZ [M+H]^+^	237.1095	C_15_H_12_N_2_O	
				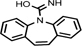

	CBZ [M+Na]^+^	259.0814	C_15_H_12_N_2_ONa	
				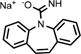

	CBZ-10,11-epoxy	253.0923	C_15_H_12_N_2_O_2_	
				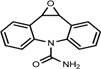

	2-hydroxy CBZ			
				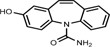

	3-hydroxy CBZ			
				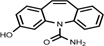

	10,11-dihydro-10,11-dihydroxy CBZ	293.0931	C_15_H_13_N_2_O_2_Na	
				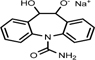

	Acridine	180.0892	C_13_H_9_N	
				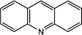

	9-hydroxy-acridine	196.0795	C_13_H_9_NO	
				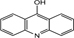

	Acridone			
				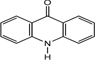

DCF	DCF [M+H]^+^	296.0217	C_14_H_10_Cl_2_NO_2_	
				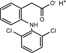

	DCF [M+Na]^+^	334.0056	C_14_H_9_Cl_2_NO_2_Na	
				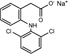

	DCF fragment	296.9910	C_13_H_9_Cl_2_NO_3_	
				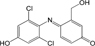

	2,6-dichlorobenzoic Na	212.9474	C_7_H_3_C_*l2*_O_2_Na	
				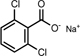

	2,4-dichlorobenzoic Na			
				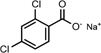

	3,5-dichlorobenzoic Na			
				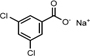

IBP	IBP [M+H]^+^	207.1352	C_13_H_18_O_2_	
				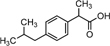

	IBP [M+Na]^+^	229.1241	C_13_H_17_O_2_Na	
				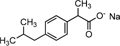

	IBP fragment	161.1337	C_12_H_17_	
				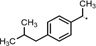

	1-hydroxyl IBP	245.1105	C_13_H_17_O_3_Na	
				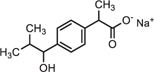

	2-hydroxyl IBP			
				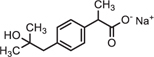

The DCF ion fragments [M+H]^+^ C14H10Cl2O2 at *m/z* 296.0217 and its sodium adduct [M+Na]^+^ C14H9Cl2O2Na at *m/z* 334.0056 were found in the liquid media test flasks and in the control batch flasks. The following could be identified only in the test batch flasks at very low peak at day 3: C13H9Cl2NO3 at *m/z* 296.9910 and the sodium adduct fragments at the mass of *m/z* 212.9474 suggested to originate from 2,6-dicholorobenzoic, 2,4-dichlorobenzoic and 3,5-dichlorobenzoic acid charring the same mass and empirical formula C7H3Cl2O2Na (Figure 5B). These DCF metabolites were present in the test flask of *A. niger* at day 10.

The IBP compound structures determined in liquid media with fungal strains were suggested to be the parent IBP C13H18O2 at *m/z* of 207.1352 and its sodium adduct C13H17O2Na at *m/z* of 229.1241. Very small amount of the intermediate ion fragments were considered to correspond to sodium adduct 1-hydroxy-IBP and 2- hydroxy-IBP (C13H17O3Na at *m/z* 245.1105 for both) as shown in Figure 5C. These last ion fragments were not visible at day 10 in liquid media test flasks inoculated with *M. circinelloides* and *T. polyzona*.

## Discussion

Preliminary tests of the fungal tolerance to the selected PhCs and enzymatic activities were conducted in solid media, to provide guidance for further experiments in liquid media and to assess PhC removal in suspensions. It has been suggested that fungal strains with the highest tolerance for contaminants could be useful for biodegradation of these contaminants in a variety of biotechnological applications (Lee et al., 2014). All of the isolated fungal strains were found to be tolerant to selected PhCs up to 1 mg/L when spiked in liquid media, either separately or in combination, based on their growth levels (mycelial mat) measured in solid and liquid media. It is also important to mention that, the isolated fungi were found to be sensitive in higher concentrations (larger than 5 mg/L) especially in combined drug preparations. Additionally, the increase in the fungal biomass concentrations (from 10 to 30% of fungal mycelium solution) seemed to enhance the tolerance index. For this reason, the experiments in wrapped ABF were performed using 30% of mycelium suspension. The results showed that, in liquid media ABF (200 mL), individual isolated fungal strains were growing in the presence of targeted PhCs in mixture up to the concentration of 1 mg/L of each PhC (while performing the simultaneous drug removal). This might be an indication of non-inhibition of the ligninolytic fungal system at this concentration. Furthermore, considering that a high concentration of PhCs has been rarely found in the environment and wastewater (Kaszubkiewicz et al., 2010; Kim et al., 2010), the isolated fungi could easily grow and produce their metabolites/enzymes under environmental conditions.

In general, and regardless of the strain, the isolated fungi appeared to be more tolerant in liquid media than in solid media. Tolerance index of over 70% were recorded in liquid media containing the combined drug concentrations of 7.5 mg/L (Figure 3), whereas in solid media (Figure 2), the isolated fungi were found to withstand PhC concentrations of 5 mg/L (FTR < 70%). Amongst the five isolated South African indigenous fungal strains, *A. niger* and *R. microsporus* demonstrated a significant drug tolerance index in solid and liquid media against the targeted PhCs (CBZ, DCF, and IBP) (Figures 2, 3). In addition, regardless of the media (solid or liquid) and working conditions (individual spike PhCs or in combination), *M. circinelloides* strain was found to be less tolerant of the three selected PhCs. Although under the present set of operating conditions, the isolated strain *T. longibrachiatum* did not exhibit a significant tolerance against the three PhCs compared to other isolated fungi, previous investigators (Lee et al., 2014) have demonstrated its tolerance against PAHs up to the concentration of 30 mg/L, such as phenanthrene (95.2%), anthracene (96.8%), pyrene (89.7%) and fluoranthene (88.1%), as well as in their mixture solution (79.4%). However, one has to bear in mind that the chemical structures of the selected PhCs differ completely from that of PAHs with exception of CBZ that can be similar to some PAHs. The current study is the first to investigate the tolerance of the five isolated fungi to selected PhCs (CBZ, DCF, and IBP). Moreover, the higher fungal tolerance (larger than 70%) to selected PhCs might be led through certain mechanisms to the gene mutations at the molecular level in fungal DNA sequences. This was reported especially in case of antifungal agents. The gene mutations were found to drive the reduction of the enzyme production (Balkis et al., 2002).

All five isolated fungal strains–one basidiomycetes (*Trametes polyzona*), two ascomycetes (*Aspergillus niger* and *Trichoderma longibrachiatum*) and two zygomycetes (*Mucor circinelloides* and *Rhizopus microsporus*) were found to be able to produce ligninolytic enzymes (Supplementary Figures 1–3). This study revealed that the production of ligninolytic enzymes is not limited only to basidiomycete fungi, members of the ascomycetes and Zygomycetes also demonstrated ligninolytic activity. The production of ligninolytic enzymes has been established in the co-culture of fungal strains *Rhizopus microsporus* KN2 and *Trichoderma* sp. KN10 by a previous investigator (Ijoma, 2016). However, among the five isolated indigenous fungi, the WRF *T. polyzona* was the only fungal strain that produced a valuable amount of all three evaluated enzymes. Furthermore, it demonstrated the production of a high concentration of Lac enzymes, regardless of the operating conditions (pH, temperature and incubation time). This finding was not surprising as this isolated fungus belongs to the phylum basidiomycetes, and is known for its enzymatic activities as reported by previous studies (Chairin et al., 2014; Teerapatsakul et al., 2016; Pinar et al., 2017). In contrast, the zygomycete fungus *R. microsporus* (belonging to the phylum zygomycota) did not show any positive reactions of the presence of Lac enzymes in its cultures. The absence of Lac enzymes in the *R. microsporus* culture has also been reported by Dolatabadi et al. (2014). On the one hand, a few studies have stated the ability of the ascomycete fungus *A. niger* (belonging to the phylum ascomycota) to produce ligninolytic enzymes, including Lac, MnP and LiP (Perez-Armendariz et al., 2010; Egwim et al., 2015). On the other hand, Aracagök et al. (2018) did not report any ligninolytic enzyme activity in *A. niger* strain used for DCF biodegradation. Only limited studies have reported ligninolytic enzyme production by *Mucor* sp. fungi. For example, Bonugli-Santos et al. (2010) have demonstrated the MnP activity exhibited by *M. racemosus* CBMAI 847. The present study reveals the ability of another *Mucor* sp., especially *M. circinelloides* to produce ligninolytic enzymes.

The effects of physicochemical parameters (pH and temperature) as well as incubation time on fungal enzymatic activity were substantial, affecting the generation and stability of crude enzymes. The fungal ligninolytic enzyme activity appeared to be higher under the following operating parameters: the pH range of 4.3–5.5; the temperature range of 30 ± 1.5–37 ± 1°C and the incubation periods from 6 to 9 days and, in particular 12 days for the Lac enzyme. The optimum conditions for enzyme production were found to be 30 ± 1.5°C, pH 4.3 and 6 days of incubation. Above and below these ideal values, the fungal enzymatic activity was shown to decline. For this reason, the pH was adjusted to around 4.3 in all the ABFs to ensure optimum production of fungal enzymes while performing PhC removal. Hence, after 5 days of spore incubation in LN-m, a certain amount of enzymes were already produced (Supplementary Figures 1–3) during fungal mycelium collection and homogenization for ABF inoculation. This might drive to the earlier biodegradation process of selected PhCs from the spiking day. The optimum pH range of 4.3 to 5.5 for enzymes production (Lac, MnP, and LiP) under the working conditions of the present study was in agreement with the findings of previous studies using other species (Schneider et al., 2018), whereas it contradicts other reports by previous investigators although the fungal species are not the same. For example, the maximum Lac activity was found for the fungus *Schizophyllum* sp. at pH 6.5 (Mahajan et al., 2015; Vijaya et al., 2017). The variation in optimum conditions for enzymatic activity seen from other studies, especially the pH of the medium, might be due to the differences in the growth media conditions as well as to the difference in fungal species, which could have led to different kinetic properties of the enzymatic activities as reported by Vijaya et al. (2017). Regarding the effect of temperature on the fungal enzymatic activity, the higher the temperature, the lower the enzymatic activity. The increase in the temperature led to the enzymatic deactivation. In addition, the enzymatic deactivation has been reported to be irreversible and leads to the change of active enzyme to a structural inactive form, and therefore the loss of the catalytic properties of crude enzymes (Puig, 2013).

The influence of incubation on enzyme synthesis by isolated fungi was also demonstrated in this investigation. After 6 to 9 days of incubation, an initial increase in enzyme concentration was followed by a progressive decline in fungal enzymatic activity. The fungal ligninolytic enzymes especially those produced by WRF, have a broad substrate specificity and have been found to be involved in the biodegradation of organo-pollutants with aromatic structural similarities to lignin, especially Lac, MnP and LiP (Pointing, 2001). In addition, there are several reports that show members of the phyla Ascomycota and Zygomycota as producers of ligninolytic enzymes, and no report is available on the simultaneous biodegradation of CBZ, DCF, and IBP. Most of the reports on these fungi including *T. polyzona* are focused on their capability to decolorize recalcitrant dyes (Fu and Viraraghavan, 2003; Cobas et al., 2013; Cerrón et al., 2015). The degradation of DCF alone was conducted using *A. niger* (Aracagök et al., 2018). Although the production of three ligninolytic enzymes and high Lac production were exhibited by the WRF *T. polyzona*, this strain did not display a better tolerance to PhCs, neither the best removal efficiency among the isolated fungi, while a number of reports raise its capacity of degrading a wide range of aromatic compounds. For example, a rapid degradation of bisphenol A and decolorization of synthetic dyes such as Congo Red, Methyl Orange and Relative Black 5 by Lac from *T. polyzona* were accounted for in the literature (Chairin et al., 2013). From the findings of the current study, a positive correlation (0.98 and 0.73) could be established between MnP production and fungal tolerance index in solid and liquid media, respectively. Unfortunately, the high tolerance of *A. niger* to selected PhCs, which was supposed to be promising of its biodegradation efficiency of drugs as predicted by Andreolli et al. (2016), was not demonstrated by this fungal strain in the ABF.

An UPLC-MS was used to determine the residual PhC concentrations in the various experiments. In order to minimize the matrix effects, the SPE technique was found to be relevant and selective for PhCs isolation from liquid media. Based on the reports of several studies that consider the optimal recovery of the sorbent when used for PhCs (López-Serna et al., 2011), and considering the preliminary assays performed using DSC-18 and HLB cartridges, the 500 mg HLB cartridges were selected. In addition, the 500 mg of sorbent cartridges were proven to produce a higher recovery compared to the 60 mg. Consequently, the sorbent amount appeared to proportionally affect the retention of PhCs. Hence, lowering the pH of the loaded solutions by 2.5 units below their pKa values of the targeted PhCs enhanced the presence of the neutral forms of analytes and to increase their interaction with the HLB sorbent for better extraction as reported by Maria Baena-Nogueras et al. (2016). The pH has been reported to control the ionization of polar analytes and therefore affects its interaction and hydrophobicity with the column sorbent (Wu et al., 2008). In addition, the HLB cartridges might have multiple retention mechanism other than hydrophobic interaction, which can be allotted to the functional groups combined to the polymeric skeleton, that have the hydrophobic and hydrophilic retentions as well as the *π-π* retention mechanisms (Wu et al., 2008). However, the reversed-phase retention mechanism of octadecyl silica in the Discovery^®^ DSC-18 SPE column appeared not to be suitable for the selected compounds, when compared to HLB. Furthermore, the almost non-existent matrix effect resulting in good recovery, precision and accuracy of the analytical method, established the analyte selectivity of the Supelco-HLB cartridges used followed by the UPLC/MS, with the Titan C18 column. Hence, the hydrophilic- lipophilic balanced (HLB) reversed-phase sorbent cartridges have been reported to be an appropriate sorbent for PhCs adsorption (Togola et al., 2014).

The selected PhCs were analyzed under positive ionized conditions and low energy, where their strong signals appeared at the mass *m/z* of 237.10, 296.02, and 229.12 for CBZ, DCF, and IBP, respectively. Thus, at this positive ionized condition, analytes were usually protonated and led to the formation of the most abundant species, ion [M+H]^+^ (CBZ) accompanied by sodium adducts [M+Na]^+^ (DCF and IBP) or with elimination of fragment [M-CHO2] (IBP). Moreover, analyte sensitivities were found to considerably decrease when performed in negative mode or in high energy. The calibration curves from SPE-UPLC/MS showed strong linearity with *r*^2^ > 0.9 for all the targeted PhCs CBZ, DCF, and IBP. The LoD and the LoQ were found to be in the same and acceptable limits reported in previous studies (Gracia-Lor et al., 2010). The reproducibility and repeatability of the SPE-UPLC/MS method generated RSD of <20% as mentioned for the acceptability of the method, in order to assess the fungal removal efficiency for selected PhCs in liquid media.

The degradation of PhCs in the ABFs produced promising results for removal by isolated fungal strains (Figures 6A–C). The results revealed that, *R. microsporus* achieved significant removal of CBZ (*p* < 0.001) at 79 and 87% after 7 and 10 days, respectively (Figure 6A); 100% DCF removal in 1 day (Figure 6B) and 85% removal of IBP removal within 7 days (Figure 6C) compared to other fungal strains. Besides being slightly removed by *T. polyzona*, CBZ demonstrated to be recalcitrant for its biodegradation by the rest of isolated fungi, with a removal recorded in the same range as the control (*p* > 0.05). The 87% removal of CBZ by *R. microsporus* after 10 days of incubation was significant for this compound as reports indicate low CBZ removal efficiency (<30%) from wastewater treatment plants (Miege et al., 2009). The DCF was found to be the most easily removed tested compound by all isolated fungi (±90% removal after 3 days). Only 10% removal of DCF was achieved within 1 day in the present study using *A. niger* strain, whereas over 80% removal was attained using the rest of the isolated fungi. The IBP was significantly removed (*p* < 0.05) by *M. circinelloides* (99%) and by *T. polyzona* (96%) within 4 days, compared to other isolated fungi. Previous experimental studies done using WRF strains such as *I. lacteus* and *P. chrysosporium* achieved 2% and no removal of CBZ, respectively. Nevertheless, these authors applied an initial concentration of 10 mg/L CBZ in their experiments which was much higher than the concentration used in the current study (Yang et al., 2013a). Around 22% of CBZ were removed using *T. versicolor* fungus within 1 day and 60% after 2 days (Hata et al., 2010a,b). Better DCF removal for *A. niger* fungus was reported by Aracagök et al. (2018) with about 70% DCF removed within 24 h, almost 100% removal after 4 h using *Trametes versicolor* fungus (Marco-Urrea et al., 2010) and complete removal after 6 days of incubation with *Phanerochaete sordida* (Hata et al., 2010a). Jelic et al. (2012) have reported <10% removal of CBZ from some conventional biological wastewater treatments. The present study showed better IBP removal than the ones reported by previous studies. Although up to 99% of IBP biodegradation was achieved by Kruglova et al. (2014) operating at laboratory scale in nitrifying activated sludge using the sequencing batch reactor system at 12°C, with a sludge retention time of 10–12 days, an immediate drop in DCF concentration was attributed to nitrifying bacteria activity, and no biodegradation in all experiments for CBZ. Almost complete IBP removal after 3 h was reported by Marco-Urrea et al. (2009) using *T. versicolor* fungus.

Although, the highest fungal tolerance index to the PhCs was recorded for *A. niger*, while the WRF *T. polyzona* demonstrated the best enzymatic activity by releasing the three ligninolytic enzymes, the better PhCs removal efficiency was exhibited by *R. microsporus*, especially for CBZ. Despite the fact that, *A. niger* produced more MnP than *R. microsporus*, the latter achieved better PhCs removal. Therefore, no correlation was found between the fungal tolerance index, the enzymatic activity and the removal efficiencies of the tested PhCs in the ABFs. Furthermore, *M. circinelloides* which exhibited a lower tolerance index to the PhCs in the combination test, displayed good DCF and better IBP removal efficiencies. This confirmed the findings by Marco-Urrea et al. (2009), which reported that ligninolytic enzymes, especially MnP and Lac failed to oxidize PhCs. This might be attributed to the reaction to other fungal metabolites.

The PhCs used in the experiments are well known to be inadequately removed by conventional wastewater treatment plants especially CBZ. A possible explanation for this inefficiency could be the presence of electron withdrawing functional groups in their molecular structures, namely amide (-CONH2) in CBZ, carboxylic (-COOH) in IBP, and carboxylic and halogen (-Cl) in DCF, which renders the compounds less susceptible to oxidative catabolism (Yang et al., 2013b). However, the low removal of CBZ could be due to the presence of the amide (azepine) group, which is a strong electron withdrawing group. Thus, the results from the current study indicated that other enzyme classes apart from the ligninolytic enzymes could have contributed to the removal of the PhCs particularly in the case of CBZ.

Decreasing pH observed in ABF (Supplementary Figure 4) can be explained by the production of fungal enzymes and secondary metabolites as discussed by Kasonga et al. (2019b), rapid microbial metabolism of added glucose and production of acidic metabolites in the ABFs. The subsequent rise in pH can be attributed to the decomposition of organic acids and CO2 stripping in the media (Voběrková et al., 2017). Additionally, the increasing pH may affect the growth of the fungal population. The decrease and sometimes the disappearance under the limit of detection of the identified analyte peaks, when working in ESI^+^ mode, did not automatically lead to the appearance of new peaks, by means of the presence of new compounds or by- products/intermediates from spiked parents. As per the setup of our experiment, the sampling period was 24 h. Therefore, some previously identified intermediate peaks produced by selected PhCs might not be found in the liquid media in the operating conditions. However, the fungal biodegradation process was evaluated by comparing the strong decrease of the selected PhC concentrations and especially the presence of a few metabolites detected in the test flasks at day 3 and day 10 (Table 3 and Figure 5). Although DCF results in the present study revealed that, suggested intermediates could be identified at very low amount at day 3 in all the test flasks, none of them was visible at day 10, when the DCF concentration was found below the limit of quantification. They were only detected in the flask inoculated with *A. niger*, where the peaks of DCF ions [M+H]^+^ at *m/z* of 296.0217 and its sodium adduct [M+Na]^+^ at *m/z* of 334.0056 still remained higher compared to the rest of the test flasks. This confirms the fact that DCF intermediates do not last in the medium, when exposed to fungi. For instance, Marco-Urrea et al. (2010) stated that, the DCF was rapidly transformed to 4’-hydroxy-DCF and 5-hydroxy-DCF by the basidiomycete WRF *T. versicolor* and these intermediates disappeared within 24 h from the medium and could not be detected after the mentioned time. The DCF fragment with accurate mass of 296.9910 was previously identified by Moreira et al. (2018) suggesting the elimination of the carboxylic acid group and structural rearrangement proposing the introduction of hydroxyl and ketone groups in the elucidated structure. Its sodium adducts isomer transformation fragments 2,4-dichlorobenzoic, 2,6-dichlorobenzoic and 3,6- dichlorobenzoic acid were earlier identified by Dodgen et al. (2014) in their acidic form. The most recalcitrant of the selected drugs CBZ has been transformed in the test flasks and almost seven (7) known ion fragments were proposed to correspond to 10,11- dihydro-10,11-dihydroxy-CBZ, acridine, 9-hydroxy-acridine, 3-hydroxy-CBZ, CBZ- 10,11-epoxide, 2-hydroxy-CBZ and acridone identified at day 3 and 10. Although their peaks were smaller compared to the peaks at *m/z* of 237.1095 and 259.0814, they could be detected in the test flasks. However, in order to identify a number of CBZ intermediates, Jelic et al. (2012) have conducted a full-scan MS of the 2 to 3 h samples collected from a *Trametes versicolor* air pulsed fluidized bed bioreactor. These authors also reported that some of the CBZ metabolites which appeared just after 2 to 4 h, may remain in the Erlenmeyer test flask medium during the experimental period of 10 days, while others emerged after 1 to 2 days of the parent spiking, including 10,11-dihydro-10,11-dihydroxy-CBZ. Apart from the IBP parent compounds ([M+H]^+^ and [M+Na]^+^), and its ion fragment at the mass *m/z* of 161.1337 suggesting an elimination of ⋅CHO2, two isomer sodium adduct fragments at accurate mass *m/z* of 245.1105 were found and proposed to match with its identified intermediates 1-hydroxy-IBP and 2-hydroxy-IBP. The metabolic pathways for the formation of IBP transformation compounds were assumed to be totally independent by previous investigator (Collado et al., 2012). The absence of most identified intermediates from the selected PhCs at day 10 could be due to the fungal biodegradation process attributed to the synergistic activity of their secreted enzymes.

The results revealed that different fungal species removed the PhCs differently. Therefore, the use of a consortium of fungal strains containing Ascomycete*s* and Zygomycetes fungi could be a solution for the simultaneous removal of different recalcitrant compounds, with different chemical structures. However, care should be taken to ensure adequate removal of very recalcitrant compounds like CBZ, which displayed significant removal after only 7 days, compared to the DCF and IBP which were almost completely removed after 2 days by all five the isolated fungal strains.

## Conclusion

The isolated fungi were found to demonstrate high tolerance to CBZ, DCF, and IBP pharmaceutical compounds. The tolerance sequence from high to low is observed for: *A. niger* and *R. microsporus* followed by the rest of the fungal species almost in the same range (*T. polyzona, M. circinelloides* and *T. longibrachiatum*). The parameters which favor the best enzymatic activities were as follows: temperature of 30 ± 1.5°C, pH range of 4.3 and an incubation time range of 6 days. The WRF *T. polyzona* is the only fungus which produces three targeted ligninolytic enzymes (Lac, LiP and MnP) in significant quantities. The highest MnP activity is exhibited by *A. niger* followed by *R. microsporus.*

In general, a positive correlation is observed between the enzymatic activity (especially for MnP production) and the fungal tolerance, while no relationship was found between the fungal tolerance and the removal efficiency, except for *R. microsporus* which displayed high tolerance and good removal of CBZ. Furthermore, no relationship between the enzyme activities of MnP, Lac and LiP and the removal efficiency for the three PhCs could not be established. The experiments done in the ABFs (with pH adjusted to around 4.3), demonstrated that, DCF was the best removed PhC, followed by IBP. The isolated strain *R. microsporus* was found to exhibit good CBZ removal followed by *T. polyzona* with considerably lower removal efficiency for the same compound. In addition, considering the level of ligninolytic enzymes and their PhC removal efficiencies, the results of this study suggest that the biodegradation ability of the fungal strains may be due to a synergistic activity of different types of released metabolites and enzymes.

Intermediate compounds for all three the PhCs were detected after 3 days of incubation. However, most of the compounds were not present in the media after 10 days of incubation. A few intermediate peaks from selected PhCs are identified especially at day 3 and are suggested to correspond to identified structures including CBZ-10,11-epoxide, 2-hydroxy-CBZ, 3-hydroxy-CBZ, 10,11-dihydro-10,11-dihydroxy- CBZ, acridine, 9-hydroxy-acridine and acridone for CBZ, the sodium adduct of 2,6- dicholorobenzoic, 2,4-dichlorobenzoic and 3,5-dichlorobenzoic acid from DCF, and 1- hydroxy-IBP and 2-hydroxy-IBP generated from IBP. Findings of the present study show that apart from the basidiomycetes, the ascomycetes and zygomycetes also display abilities to biodegrade emerging pollutants such as PhCs and to produce the ligninolytic enzymes (Lac, MnP, and LiP).

## Data Availability Statement

The original contributions presented in the study are included in the article/Supplementary Material, further inquiries can be directed to the corresponding author/s.

## Author Contributions

MM, IK, and MC conceived, designed, and funded the project and reviewed the manuscript. TK performed the experiments and drafted the manuscript. TK, MM, MC, and IK analyzed the data. All authors contributed to the article and approved the submitted version.

## Conflict of Interest

The authors declare that the research was conducted in the absence of any commercial or financial relationships that could be construed as a potential conflict of interest.

## Publisher’s Note

All claims expressed in this article are solely those of the authors and do not necessarily represent those of their affiliated organizations, or those of the publisher, the editors and the reviewers. Any product that may be evaluated in this article, or claim that may be made by its manufacturer, is not guaranteed or endorsed by the publisher.
