# Supplementary material for: Assessing the Fungal Simultaneous Removal Efficiency of Carbamazepine, Diclofenac and Ibuprofen in Aquatic Environment
Source: Front Microbiol. 2021 Dec 13;12:755972. doi: 10.3389/fmicb.2021.755972 (PMC8710540; doi:10.3389/fmicb.2021.755972)
Supplement: Supplementary file 1 [file Data_Sheet_1.docx]

**Supplementary results**

**1 Assessment of the fungal ligninolytic enzymatic activity**

### *1.1 Enzymatic activity assay in solid medium*

Table S1 displayed the formation of colour zone in indicator plates demonstrating the production of oxidative enzymes, most probably the targeted enzymes: Lac, LiP and MnP. The formation of slight (+) or intense (++) colour zone in the indicator plates supplemented with mediating substrates ABTS, VA and 2,6-DMP was indicating the production of ligninolytic enzymes Lac, LiP and MnP. Indicator plates supplemented with ABTS have exhibited fungal growth and colour production revealing of Lac production, except for *R. microsporus.* Fungal growth or any specific colour production were not observed in the indicator plates supplemented with VA at 25 mL/L. Thus, when VA concentrations was decreased at 10 mL/L and 1 mL/L, fungi were growing in indicator plates without any specific colour zone production for LiP production within 7 days of incubation. However, a slight appearance of colour zone was observed in *T. polyzona* and *R. microsporus* plates, occurring when the growth was allowed to extend for 14 days. The method seemed not to work in the case of indicator plates supplemented with VA. The 2,6-DMP containing plates of all isolated fungal species have demonstrated a fungal growth and specific colour zone formation revealing of MnP enzyme production.

### *1.2 Enzymatic activity assay in liquid medium (LN-m)*

The average values of fungal enzyme activities assayed for Lac, LiP and MnP demonstrated that, MnP production was higher (*p* < 0.05) followed by LiP and Lac, except the WRF *T. polyzona* which exhibited high Lac enzyme activity (*p* < 0.001) than MnP and LiP. Apart from the WRF *T. polyzona* which has demonstrated the production of three targeted ligninolytic enzymes in the working conditions, the Ascomycetes fungi *A. niger* and *T. longibrachiatum* as well as Zygomycetes fungi *M. circinelloides* and *R. microsporus* were found producers of ligninolytic enzymes of biotechnological interest, specifically MnP and LiP.

**Assessing the effect of pH on enzymatic activity in LN-m**

Figure S1 demonstrated the effect of pH on ligninolytic enzymes production in LN-m after 5 days of incubation at 30 ± 1.5 °C in absence of targeted PhCs. The enzymatic activity was found in general to be high at initial pH range between 4.3 and 5.5 and decreased strongly when initial pH was low between 2.3 and 3.1 or beyond 5.5. The lowest enzyme activity was observed for MnP and nothing for LiP and Lac, except *T. polyzona* which showed a slight Lac activity at initial pH range of 2.3 - 3.1. Beyond pH 5.5, MnP activity was observed for all the isolated fungi. The optimal pH value for MnP production was recorded at 4.3 and 5.5 for *A. niger* (211.14±8.22 U/L, *p* < 0.05) and only 4.3 for the rest of fungal strains. It was followed by *R. microsporus* (159.88±9.12 U/L)*, M. circinelloides* (131.24±5.01 U/L), *T. longibrachiatum* (100.23±4.12 U/L) and *T. polyzona* (80.19±4.51 U/L)*.* The pH of 4.3 appeared also to be the optimum for Lac production with *T. polyzona* (199.18±8.15 U/L, *p* < 0.001) compared to other isolated fungi, for which slight activity was observed in flasks inoculated with *A. niger* (2.2±0.01 U/L)*, T. longibrachiatum* (0.08 U/L) and *M. circinelloides* (1.12±0.02 U/L)*.* The optimum activity for LiP was observed at 5.5 with *T. polyzona.* In addition, the fungal enzymatic activity was still observed over pH values slightly out of the optimum range.

**Assessing the effect of temperature on enzymatic activity in LN-m**

Regarding the effect of temperature on ligninolytic enzymatic activity (Figure S2), the enzyme production appeared to be better in the temperature range of 25±1 °C to 37±1 °C. The enzyme production generally decreased above 37±1 °C and below 28±1 °C. Although the highest MnP production was accomplished by *A. niger* (225.63±8.95 U/L) and the highest LiP production was achieved with *T. polyzona* (75.12±1.35 U/L) at 37±1 °C, a temperature of 30±1.5 °C seemed to be the optimum for the three ligninolytic enzyme production with highest Lac produced by *T. polyzona* (199.18±8.15 U/L) and MnP and LiP found in all fungal cultures. In general, the temperature was found to affect significantly (*p* < 0.05) the ligninolytic enzyme production. Taking into account that, the incubation time was of 5 days for different temperatures (20±1.5 °C, 25±1 °C, 30±1.5 °C, 37±1 °C and 45±1 °C), it appeared that the enzyme production was function of temperature and incubation time. The enzyme half-life might be shorter at higher temperature for the same incubation time, which was a result of lower activity in the medium.

**Assessing the effect of incubation time on enzymatic activity in LN-m**

The Figure S3 displayed the effect of the incubation time of isolated fungal strains in the production of enzymes. The highest level of MnP activity (239.96±6.26 U/L) was achieved on day 6 with *A. niger*, while for Lac and LiP activities the fungal strain *T. polyzona* produced a maximum activity of 200.18±8.15 U/L and 65.13±1.01 U/L respectively for 6 days old cultures. However, the Lac activity increased in 12 days old cultures when compared to the 9 days old cultures of *T. polyzona*. The incubation time range from 6 to 12 days was found to give better enzyme production with an optimum on day 6.

**List of figures**


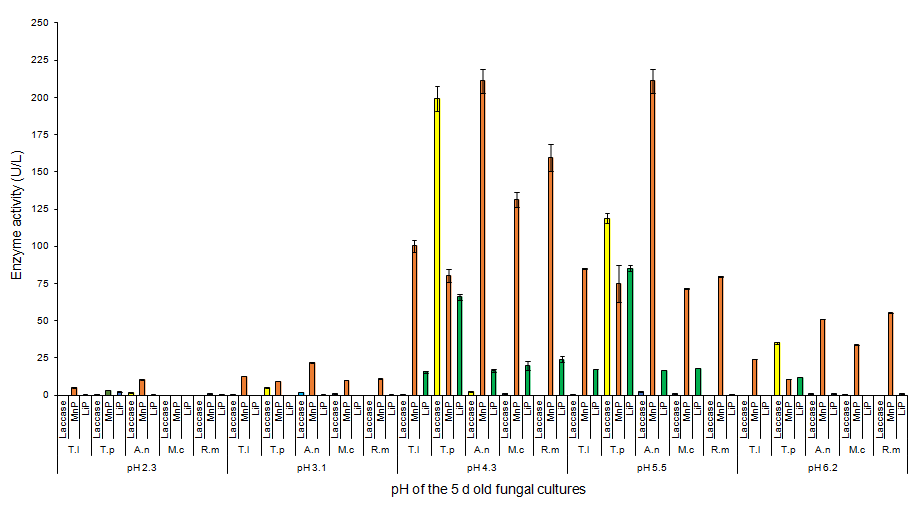


**Figure S1:** Assessing the pH effect in ligninolytic enzymatic activity assay


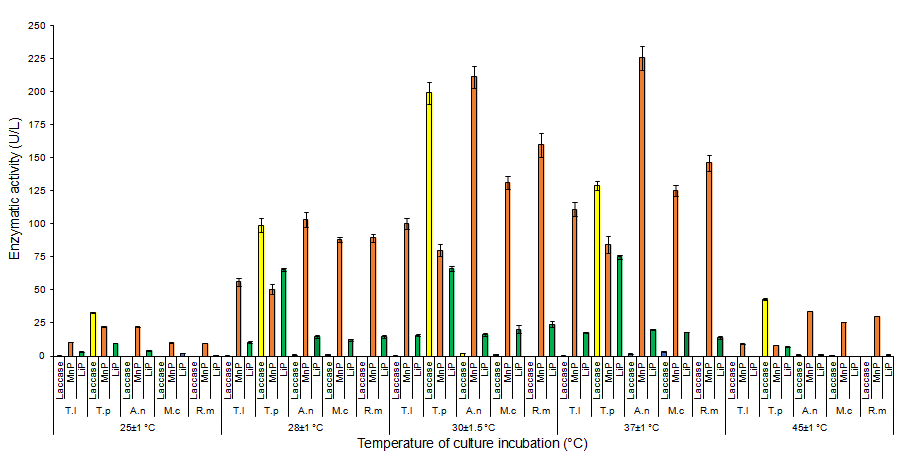


**Figure S2:** Evaluation of incubation temperature effect in ligninolytic enzymatic activity assay


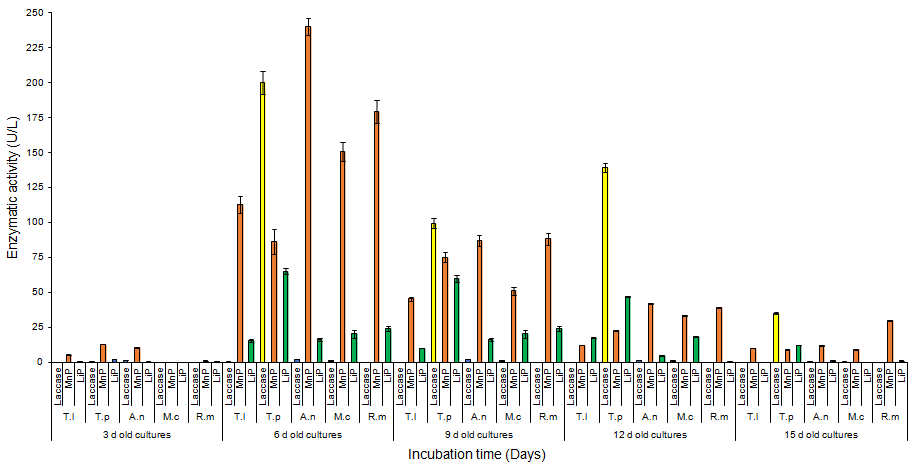


**Figure S3:** Effect of incubation time in ligninolytic enzymatic activity assay

**Figure S4.** Adjusted pH of aerated flasks throughout the experimental study.


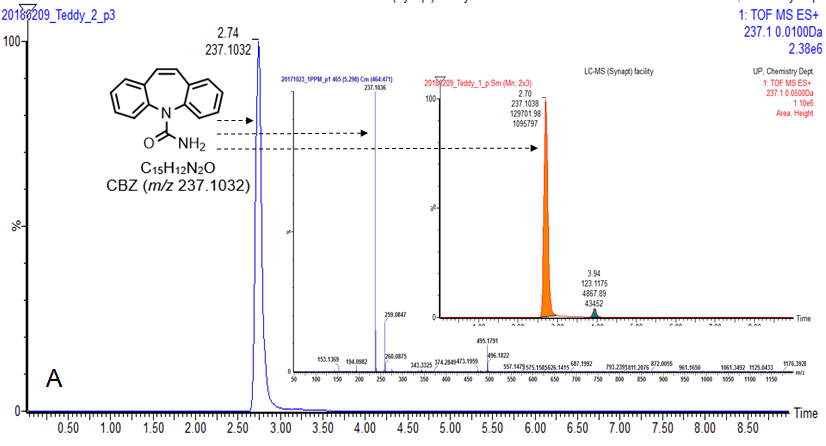


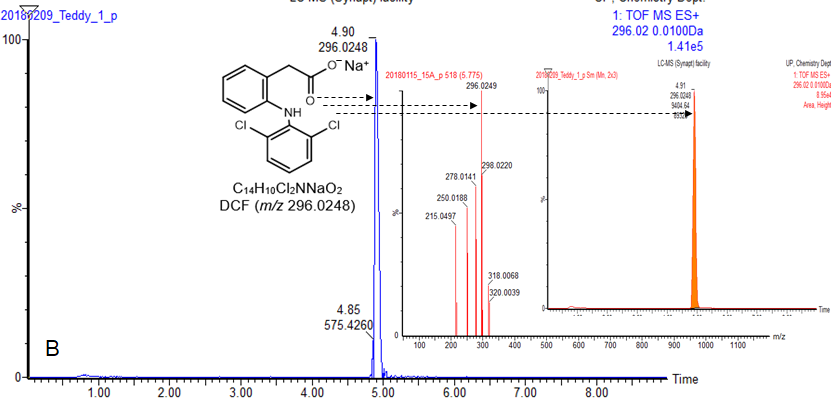


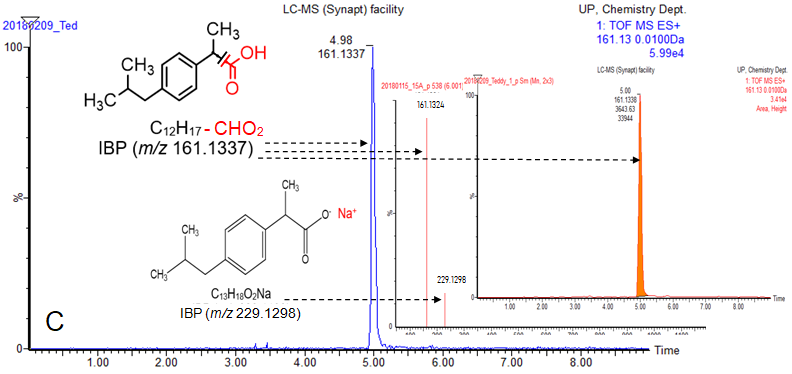


**Figure S5.** Selected UPLC-(+)-ESI-QToF-MS chromatograms and production spectrum of (A): CBZ, (B): DCF and (C): IBP

**Figure S6.** PhC calibration curves (CBZ, DCF and IBP)

**List of Tables**

**Table S1**. The fungal enzymatic activity essay in solid medium

| ID  Number | Reagents | ABTS | VA | 2,6-DMP |
| --- | --- | --- | --- | --- |
|  | Targeted enzyme | Lac | LiP | MnP |
| 1 | *T. longibrachiatum* | + | - | + |
| 2 | *T. polyzona* | ++ | + | + |
| 7 | *A. niger* | + | - | ++ |
| 8 | *M. circinelloides* | + | - | ++ |
| 11 | *R. microspores* | - | + | ++ |

(+) slight specific colour zone observed, (++) intense specific colour zone, (-) absence of specific colour zone.
